# Postural Stability in Children with Cerebral Palsy

**DOI:** 10.3390/jcm13175263

**Published:** 2024-09-05

**Authors:** Andrzej Szopa, Małgorzata Domagalska-Szopa

**Affiliations:** 1 Department of Physiotherapy, Medical University of Silesia in Katowice, 40-752 Katowice, Poland; 2 Neuromed, Rehabilitation and Medical Center, 40-698 Katowice, Poland; 3 Department of Developmental Age Physiotherapy, Medical University of Silesia in Katowice, 40-752 Katowice, Poland

**Keywords:** cerebral palsy, body weight distribution, neurology, paediatric

## Abstract

**Background:** A lack of postural control is one of the key problems in children with cerebral palsy (CP). The goals of the present study were to assess static postural stability in children with mild CP using a force platform compared to that of typically developing peers and to identify differences in static stability between children with hemiplegic and diplegic CP. **Methods:** This study included 45 children with hemiplegic CP and 45 children with well-functioning diplegic CP (Gross Motor Function Classification System; GMFCS scores between I and II) who were patients of local paediatric rehabilitation centres. The testing procedure included two interrelated parts: (1) the analysis of the body weight distribution and (2) the posturometric test (the centre of pressure; CoP measurements) using the force platform. **Results:** The results of the present study show that children with CP, compared to their TD peers, demonstrated significantly higher values for all of the analysed indexes of postural stability. The obtained results indicate differences in disorders of static postural stability between children with hemiplegic and diplegic CP. Compared to their TD peers, children with hemiplegic CP showed greater body weight asymmetry between the affected and unaffected sides of the body and greater CoP sway in the medial–lateral direction. In contrast, children with diplegic CP exhibited greater CoP displacements in the anterior–posterior direction. **Conclusions:** The findings of the present study show that (1) children with CP have increased static postural instability compared to their TD peers and (2) children with diplegic CP exhibit weaker mediolateral stability in standing, whereas children with hemiplegic CP show reduced anterior–posterior stability.

## 1. Introduction

The current definition of cerebral palsy (CP) is a postural and movement disorder resulting from a brain lesion or a developmental disorder of the immature brain [1]. These disorders appear in the early stage of psychomotor development and manifest themselves in the form of delay, abnormal postural patterns, and pathological motility, the severity of which increases with a child’s physical and motility development. The most visible outcomes of postural control are the alignment of the body segments, body orientation relative to the gravitational force (a postural pattern), and control of the body position, i.e., maintaining the centre of pressure (the CoP) over the base of support (the BoS) under a static or dynamic condition [2]. Static postural stability control consists of constant minor motions of a body—forward and backward (the anterior–posterior direction; AP) and side-to-side directions (the medial–lateral direction; ML). Dynamic postural balance control refers to maintaining postural stability and orientation with the CoP over the BoS during the intentional (i.e., while the body parts are in motion) or the unintentional movement (e.g., when the ground is in motion).

Postural control is the collective result of coordinated actions of the central nervous system, which is based on the visual, haptic, and vestibular systems, and requires the coordination of numerous muscle synergies simultaneously engaging over multiple joints and segments of the body [2].

The literature indicates that children diagnosed with CP show deficiencies in steady-state control [3,4,5] and dynamic control [6,7,8,9,10,11,12,13,14]; however, different study protocols and various research tools were used [15]. For this reason, there is no consensus in the subject literature regarding the occurrence of postural stability disorders in children with CP.

Although some studies have shown that these disorders depend on their functional status [10,11,12,13,14], there needs to be more studies on the differences in standing stability between diplegic and hemiplegic patients. Only one study has suggested that children with spastic diplegia exhibit worse postural balance control ability compared with spastic hemiplegia. However, this finding is based on a small sample size, i.e., 7 children with spastic diplegia and 16 with spastic hemiplegia, and covers a wide age range of participants (from 7 to 16 years of age) [16].

Given the above findings, this study aimed to assess the postural stability of children with mild CP compared to their healthy peers and to compare postural stability between children with hemiplegic and diplegic CP. However, the most important goal was identifying the differences in static postural stability disorders in children with hemiplegic and diplegic CP. The research hypotheses were as follows: (1) Compared to healthy peers, children with mild CP exhibit a greater amount of CoP sway. (2) Children with spastic diplegia exhibit worse postural stability compared with children with spastic hemiplegia. (3) Postural stability disorders in children with hemiplegic and diplegic CP are differentiated.

## 2. Materials and Methods

This study was approved by the Bioethics Committee of the Medical University of Silesia in Katowice (NN-013-350/I/03/04). The guardians of all the participants gave their written and informed consent for the children to participate in the study prior to data collection. All work was performed by the Code of Ethics of the World Medical Association (in accordance with the Declaration of Helsinki). An a priori sample size analysis was performed to detect an effect size with the α value of 0.05 and to determine the power of the study (80–152% participants). Based on these calculations, approximately 40 participants were recruited for each study group.

### 2.1. Subjects

This study included 45 children (17 girls and 28 boys) with spastic hemiplegia and 45 children (22 girls and 23 boys) with spastic diplegia. All participants with CP were successively recruited one by one from groups of well-functioning patients of local paediatric rehabilitation centres and had Gross Motor Function Classification System (GMFCS) scores between I and II [17].

All subjects met the following inclusion criteria: (1) they were diagnosed with spastic hemiplegia or diplegia; (2) they were at least 7 years old and no older than 12 (to minimise the incidence of patterns of postural instability at a younger age and during the adolescence period); (3) they were able to maintain the standing position without additional support for 1 min; and (4) they were able to follow the verbal instructions. The exclusion criteria were as follows: (1) a history of uncontrolled seizures or vestibular dysfunction; (2) previous surgical procedures; and (3) pharmacological treatment and spasticity management for six months before the evaluation.

The control group (controls) comprised 45 typically developing (TD) peers (18 girls and 28 boys). Baseline data for all participants are shown in Table 1. There were no significant differences in participants’ baseline data between the control, spastic diplegia, and spastic hemiplegia groups (Table 1).

### 2.2. Testing Procedure

The testing procedure included two interrelated parts: (1) the analysis of the body weight distribution (BWD) and (2) the posturometric test (the CoP measurements). At first, the BWD between the sides of the body was measured, and then the posturometric test of the CoP was performed with open eyes. Both results were obtained by using the force platform (PDM Multifunction Force Measuring Plate, Zebris, Isny, Germany). During the tests, the participants stood barefoot on the force platform in a natural position, with their arms hanging loosely at their sides. The BWD measurement included three trials, and the mean value of the BWD between the right and left sides of the body for the controls and children with diplegia, as well as the BWD between the affected and unaffected sides of the body for children with hemiplegia, were used in the future statistical analysis. Posturometric tests included three trials lasting 30 s with a 30 s pause between them, and the average values of obtained the results were statistically analysed.

The analysis covered the following posturometric parameters: (1) the BWD between the right and left sides of the body; (2) stability indexes based on the CoP displacement: the mean value of points with x-coordinates (MCoCx) (the medial–lateral direction of the CoP), the mean value of points with y-coordinates (MCoCy) (the posterior–anterior direction of the COP), the sway path length of the CoP (SPL); and (3) stability indexes based on the CoP area: the area of the centres of pressure (AoE, calculated from the CoP shifts in such a way that 95% of the data are within the ellipsoid and 5% are outside), the width of the ellipse (WoE, the lateral sway path of the CoP), and the height of the ellipse (HoE, the posterior sway path of the CoP) (Table 2).

### 2.3. Data Collection and Analysis

The BWD and CoP shifts were the base for calculating the following indexes of stability (Table 1):

(1) The BWD between the sides of the body while standing;
(2) The indexes of stability based on the CoP shifts while standing;
(3) The indexes of stability based on the surface area of the CoP while standing.

### 2.4. Statistical Analysis

The normality of the distribution of analysed parameters was assessed using skewness, kurtosis, and the Shapiro–Wilk test. Normally distributed variables were summarised as mean values and standard deviations; non-normally distributed variables were presented as medians and ranges.

The Index of Asymmetry (IA) was calculated for all participants based on the BWD between body sides according to the formula IA = NP − P/NP + P, where NP = the nonparetic/dominant side percentage load distribution, and P = the paretic/nondominant side percentage load distribution [18].

The non-parametric ANOVA and Mann–Whitney test were performed on all independent variables and between-group factors. The alpha level of 0.05 was chosen as the cut-off value for all statistical comparisons, and the least significant difference (LSD) post hoc comparison was performed. The Statistical Software Package TIBCO (version 13.3 PL) was used for statistical analysis purposes. For the above analyses, a sensitivity analysis was performed in the G* Power 3.1.9.4 programme, assuming 95% of the statistical test power, α = 0.05, and N = 45.

## 3. Results

The next part of the article includes the obtained results, such as the following: (1) the asymmetry of the body weight distribution presented relatively to the overall weight distribution while standing between the right and left sides of the body for the controls and children with diplegia and between the affected and unaffected sides of the body for children with hemiplegia; (2) the indexes of stability of the hemiplegics, diplegics, and controls based on the CoP displacement and the CoP area while standing.

Table 3 presents the descriptive statistics for the indexes of postural stability in children with CP and the control group as well as two examined subgroups: children with CP (hemiplegia and diplegia) and their TD peers.

A comparison of the results between the children with CP showed that, in general, children with CP demonstrated statistically significantly higher values for all analysed indexes of postural stability (Table 4).

A comparative analysis of the stability indexes between subgroups showed statistically significant differences in almost all analysed indexes of postural stability between the controls and children with hemiplegia and diplegia (Table 5).

The analysis of the BWD asymmetry revealed that, in comparison with the other two groups, children with hemiplegia showed an extremely high IA value (Table 3). However, there were no significant differences between the controls and children with diplegia (Table 5).

Interestingly, children with diplegia have shown values approximately three times higher for the sway path length of the CoP (SPL) and values two times higher for the lateral sway path of the CoP (the width of the ellipse; WoE) and the area of the centre of pressure (AoE) than the controls. Moreover, children with hemiplegia have demonstrated values approximately three times higher for the lateral sway path of the CoP (the width of the ellipse; WoE) and values two times higher for the sway path length of the CoP (SPL) than the controls (Table 5). However, no significant differences existed between the groups of children with CP (Table 5).

The basic indexes of stability based on the medial–lateral and anterior–posterior displacement of the CoP (respectively, WoE and HoE) and the basic indices of stability based on the surface area of the CoP (AoE) were higher for children with diplegia than for children with hemiplegia. Furthermore, the post hoc analysis (the NIR test) revealed that all of the above-mentioned differences proved to be statistically significant (Table 5).

## 4. Discussion

The first aim of this study was to answer the question of whether highly functional children (GMFCS level I or II) with CP show symptoms of postural stability disorders. The obtained results show that the basic indexes of static postural stability based on CoP displacements and those based on the surface area of the CoP were significantly higher (i.e., worse) for children with CP than for their TD peers. In comparison to the TD peers, the features of postural instability were manifested in children with both types of CP. The CoP sway path length (SPL) was three times longer in children with diplegia and twice as long in children with hemiplegia compared to the controls. However, although the differences in the mean values of the sway path length between children with hemiplegic and diplegic CP were major, they were not statistically significant. The sway path length represents the total distance that the CoP of the subject covered during the test in a standing position [19]. Extending that distance proves that static postural stability is impaired [19]. With regard to the sway path length, the significantly worse results for children with both types of CP compared to their healthy peers unambiguously prove the occurrence of disorders of static postural stability in these children.

The parameters that significantly distinguished children with CP and both types of CP from their TD peers were greater CoP displacements in both the medial–lateral and the anterior–posterior directions and the surface of the CoP area in children with CP. These results unambiguously prove that static postural stability disorders occur in children with mild CP. This is consistent with the results of previous studies, which reported that children with CP compared with their TD peers generate larger profiles of postural sway while standing, such as a higher sway path length of the CoP, greater CoP displacements, and a greater sway area [3,4,5,12,20,21,22,23,24,25,26].

Despite the above, several previous studies reported that postural stability disorders depend on the functional status of children with CP [10,11,12,13,14], which could indicate poorer stability in children with diplegia; our results only partially confirm this. The poorer ability to stabilise in children with diplegia compared to children with hemiplegia was confirmed by the significantly higher values of the CoP displacements in the anterior–posterior direction (HoE) and greater surface area of the CoP (AoE). On the other hand, as already mentioned, the differences in mean values of the sway path length (SPL) between children with hemiplegic and diplegic CP were not statistically significant. Moreover, although both groups of children with CP demonstrated higher CoP displacements in the medial–lateral direction compared to their TD peers, the width of the ellipse (WoE) of children with hemiplegia was significantly larger than that in children with diplegia.

The picture of postural stability disorders in children with CP is complemented by the results of the analysis of the differences between children with hemiplegic and diplegic CP. The main posturometric parameters distinguishing children with hemiplegia from children with diplegia were CoP displacements in the mediolateral and anterior–posterior directions.

The primary differences in disorders of static postural stability between children with hemiplegic and diplegic CP may be summarised as follows:

- Children with hemiplegic CP showed greater body weight asymmetry between the affected and unaffected sides of the body and greater CoP sway in the medial–lateral direction compared to their TD peers.
- Children with diplegic CP exhibited greater CoP displacements in the anterior–posterior direction than their TD peers.

Given that a lack of postural stability has a direct impact on the ability to perform everyday activities [27], evaluating postural control in children with CP is crucial for quantifying the lack of stability, determining the nature of prevalent disorders, targeting therapeutic interventions based on that information, and monitoring and measuring progress during rehabilitation.

The present study’s value is undoubtedly due to the tested groups (45 participants with hemiplegia and 45 participants with diplegia) and the equally numbered control group (45 TD peers), as well as the evaluation of stability by means of the force platform in clinical conditions, i.e., in accordance with the golden standard for the measurements of steady-state stability, which provides the most accurate evaluation [15].

However, our study has some limitations, primarily the lack of evaluation of postural stability with closed eyes. This was because some of the subjects (mostly those at younger ages) were not able to complete that task. Furthermore, due to the applied research tool, which required maintaining the standing position independently, the study was limited to the group of children with mild CP, excluding those with higher degrees of disability (GMFCS levels III-V). Due to the dependence of postural stability disorders on the functionality level of a child with CP, which has been raised in some previous studies, future research should be extended by the examination with closed eyes and include children with CP with a lower level of functionality. Another limitation may be that there is no information on one physiotherapy intervention for children with CP (i.e., type and intensity), which may marginally disrupt our results.

## 5. Conclusions

The findings of the present study concluded that (1) children with CP have increased static postural instability compared to their TD peers and (2) the nature of disorders of static postural stability in children with hemiplegic and diplegic CP is varied: children with diplegic CP exhibit weaker mediolateral stability while standing, whereas children with hemiplegic CP show reduced anterior–posterior stability.

## Figures and Tables

**Table 1:** The characteristics of the studies and control groups. A between-group comparison of the demographic characteristics.

Parameters	Hemiplegia (N = 45)	Diplegia (N = 45)	Control (N = 45)	K-W; *p*-Value
	M ± SD; Min–Max	M ± SD; Min–Max	M ± SD; Min–Max	
Age (y)	9.87 ± 2.05; 7–12	9.84 ± 2.04; 7–12	9.22 ± 1.86; 7–12	2.97; 0.22
Height (cm)	135.93 ± 13.21; 108–160	134.02 ± 13.59; 112–160	132.60 ± 12.18; 112–155	1.36; 0.50
Weight (kg)	35.62 ± 11.87; 18–61	34.75 ± 10.08; 19–56	31.73 ± 9.93; 18–52	3.26; 0.19
BMI	18.64 ± 3.39; 13.23–27.30	18.90 ± 2.63; 13.88–23.31	17.54 ± 2.86; 13.22–24.74	5.25; 0.07
Girls; N (%)	17 (37.78%)	22 (48.89%)	18 (40%)	χ^2^ = 1.27; 0.52
Boys; N (%)	28 (62.22%)	23 (51.11%)	28 (60%)	
GMFCS I; N (%)	19 (42.22%)	21 (46.67%)	0 (0%)	χ^2^ = 0.18; 0.67
GMFCS II; N (%)	26 (57.78%)	24 (53.33%)	0 (0%)	

M, mean; SD, standard deviation; min-max, minimum–maximum; K-W, ANOVA via Kruskal–Wallis; y, year.

**Table 2:** The postural stability indices are based on the WB and CoP shift distribution during standing.

Indices based on the WB distribution	
IA	The asymmetry ratios were calculated using the formulae IA = WBR-WBL (for children with diplegia) and IA = WBH-WBN (for children with hemiplegia). The IA is a dimensionless value calculated by the difference in the WB values of the right ^®^ or affected (H) side from those of the left (L) or unaffected (N) side. As such, values of IA = 0 represent total WB symmetry in each position, values of IA > 0 represent WB asymmetries towards the affected side, and values of IA < 0 represent WB asymmetries towards the unaffected side.
Stability indices based on the CoP	
MCoCx	mean of points with x-coordinates (medial–lateral direction of CoP)
MCoCy	mean of points with y-coordinates (posterior–anterior direction of CoP)
SDx	standard deviation of x’
SDx	standard deviation of x’
SPL [cm]	sway path length of CoP (SP)
Stability indices based on the surface area of the CoP	
WoE [cm]	width of ellipse (lateral sway path of CoP)
HoE [cm]	height of ellipse (posterior sway path of CoP)
AoE [cm^2^]	area of centres of pressure (calculated from CoP shifts in such a way that 95% of data are within ellipsoid and 5% are outside).

**Table 3:** A summary of asymmetry (IA) and the selected indexes of static postural stability of the children with hemiplegia (N = 45), children with diplegia (N = 45), children with hemiplegia and diplegia (N = 90), and controls (N = 45).

Parameters	Hemiplegia	Diplegia	Hemi/Dipl	Controls	Hemiplegia	Diplegia	Hemi/Dipl	Controls
	M ± SD; Min–Max	M ± SD; Min–Max	M ± SD; Min–Max	M ± SD; Min–Max	S-W; *p*-Value			
IA	0.26 ± 0.10; 0.12–0.48	0.11 ± 0.09; 0.02–0.38	0.19 ± 0.12; 0.02–0.48	0.08 ± 0.05; 0–0.22	0.95; 0.08 *	0.84; 0.00	0.93; 0.00	0.93; 0.01
MCoCx	17.35 ± 2.11; 11.02–21.49	14.73 ± 2.96; 7.7–19.19	16.58 ± 2.67; 7.7–21.49	17.81 ± 2.16; 14.43–23.45	0.97; 0.36 *	0.96; 0.11 *	0.96; 0.03	0.97; 0.33 *
MCoCy	22.58 ± 2.40; 17.15–27.53	23.67 ± 2.07; 18.24–29.27	22.90 ± 2.16; 17.15–29.27	23.13 ± 1.15; 20.72–25.63	0.97; 0.43 *	0.96; 0.11 *	0.95; 0.01	0.98; 0.72 *
SDx	0.63 ± 0.25; 0.28–1.16	0.88 ± 0.53; 0.05–2.16	0.76 ± 0.44; 0.05–2.16	0.62 ± 0.23; 0.27–1.13	0.92; 0.01	0.96; 0.09 *	0.93; 0.00	0.96; 0.17 *
SDy	0.64 ± 0.25; 0.15–1.19	0.71 ± 0.29; 0.34–1.32	0.68 ± 0.30; 0.15–1.98	0.45 ± 0.21; 0.15–1.12	0.97; 0.29 *	0.92; 0.00	0.93; 0.00	0.91; 0.00
SPL	115.73 ± 23.81; 72.6–162.42	149.10 ± 52.51; 74.17–245.97	132.42 ± 43.88; 72.6–245.97	58.76 ± 28.23; 17.91–105.68	0.96; 0.19 *	0.94; 0.03	0.92; 0.00	0.93; 0.01
WoE	7.57 ± 2.74; 3.36–13.77	5.54 ± 2.35; 1.43–10.01	5.82 ± 2.79; 1.43–13.77	2.88 ± 1.34; 0.94–5.64	0.96; 0.09 *	0.97; 0.23 *	0.95; 0.00	0.92; 0.00
HoE	4.30 ± 2.24; 2.01–11.42	8.67 ± 2.26; 3.81–12.33	6.21 ± 3.03; 2.01–12.33	4.41 ± 1.55; 1.94–7.61	0.84; 0.00	0.96; 0.16 *	0.93; 0.00	0.97; 0.20 *
AoE	15.92 ± 10.03; 1.61–38.77	21.59 ± 11.09; 4.63–42.45	18.76 ± 10.90; 1.61–42.45	9.77 ± 6.36; 1.56–27.57	0.89; 0.00	0.94; 0.02	0.92; 0.00	0.91; 0.00

M, mean; SD, standard deviation; min-max, minimum–maximum; S-W, Shapiro–Wilk test; Hemi/Dipl, children with hemiplegia and diplegia; * *p* > 0.05.

**Table 4:** The indexes of static postural stability of the children with CP (hemiplegia and diplegia (N = 90) and control group (N = 45) and between-group differences and the results of the non-parametric Mann–Whitney test.

Parameters	Hemiplegia and Diplegia	Control Group	M-W
	Me; Q1–Q3; IQR	Me; Q1–Q3; IQR	Z; *p*-Value
IA	0.17; 0.00–0.30; 0.22	0.08; 0.04–0.12; 0.08	−5.19; 0.00 *
MCoCx	16.87; 14.87–18.27; 3.40	17.71; 16.06–18.97; 2.91	2.24; 0.02 *
MCoCy	23.03; 21.94–23.90; 21.98	23.36; 22.41–23.77; 1.36	0.53; 0.59
SDx	0.74; 0.43–0.94; 0.51	0.58; 0.45–0.75; 0.30	−1.73; 0.08
SDy	0.65; 0.42–0.87; 0.45	0.39; 0.28–0.56; 0.28	−4.81; 0.00 *
SPL	124.24; 96.71–155.69; 58.98	81.31; 55.31–104.90; 49.59	−6.41; 0.00 *
WoE	5.36; 3.67–7.37; 3.7	2.60; 1.79–3.87; 2.08	−6.31; 0.00 *
HoE	5.59; 3.46–8.67; 5.21	4.32; 3.26–5.4; 2.14	−3.16; 0.00 *
AoE	15.20; 10.5–28.16; 17.66	9.29; 4.75–12.16; 7.41	2.24; 0.00 *

Me, median; Q1-Q3, lower and upper quartiles; IQR, interquartile range; M-W, Mann–Whitney test; * statistically significant values, *p* < 0.05.

**Table 5:** The indexes of static postural stability of the children with hemiplegia (N = 45), children with diplegia (N = 45), and controls (N = 45) and between-group differences. A non-parametric ANOVA was carried out.

Parameters	Hemiplegia	Diplegia	Controls	K-W; *p*-Value; Post Hoc LSD
	Me; Q1–Q3; IQR	Me; Q1–Q3; IQR	Me; Q1–Q3; IQR	
IA	0.26; 0.20–0.34; 0.14	0.08; 0.06–0.12; 0.06	0.08; 0.04–0.12; 0.08	66.67; 0.00 * ^a,b^
MCoCx	17.31; 16.26–18.28; 2.02	14.48; 13.34–17.49; 4.15	17.71; 16.06–18.97; 2.91	26.64; 0.00 * ^a,c^
MCoCy	22.41; 21.48–24.18; 2.70	23.71; 22.43–24.3; 1.87	23.36; 22.41–23.77; 1.36	6.51; 0.04 * ^a^
SDx	0.62; 0.42–0.80; 0.38	0.79; 0.66–1.2; 0.54	0.58; 0.45–0.75; 0.30	9.79; 0.01 * ^a,c^
SDy	0.64; 0.40–0.86; 0.46	0.68; 0.42–0.93; 0.51	0.39; 0.28–0.56; 0.28	23.85; 0.00 * ^b,c^
SPL	115.02; 96.71–135.29; 38.58	143.14; 108.79–177.49; 68.7	55.63; 34.05–81.34; 47.29	71.03; 0.00 * ^b,c^
WoE	7.11; 5.57–9.36; 3.79	5.36; 4.12–70; 2.88	2.60; 1.79–3.87; 2.08	62.62; 0.00 * ^a, b,c^
HoE	3.46; 2.58–5.54; 2.96	8.76; 7.29–10.42; 3.13	4.32; 3.26–5.40; 2.14	62.17; 0.00 * ^a,c^
AoE	12.91; 9.57–21.07; 11.50	19.93; 12.02–29.92; 17.90	9.29; 4.75–12.16; 7.41	30.18; 0.00 * ^a,b,c^

Me, median; Q1–Q3, lower and upper quartiles; IQR, interquartile range; K-W, ANOVA via Kruskal–Wallis test; * statistically significant values; post hoc LSD, least significant difference test (^a^ hemiplegia vs. diplegia, ^b^ hemiplegia vs. control, ^c^ diplegia vs. control).

## Data Availability

The datasets generated during and/or analysed during the current study are not publicly available because the data are part of an ongoing study, but they are available from the corresponding author (aszopa@sum.edu.pl).
